# Room-Temperature Synthesis of Thioether-Stabilized Ruthenium Nanocubes and Their Optical Properties

**DOI:** 10.1021/acs.langmuir.2c02645

**Published:** 2023-02-01

**Authors:** Clara P. Adams, Chartanay D. J. Bonner, Gayani Pathiraja, Sherine O. Obare

**Affiliations:** † Central Piedmont Community College, 1201 Elizabeth Avenue, Charlotte, North Carolina28204, United States; ‡ Department of Chemistry, Western Michigan University, 1903 W. Michigan Ave.Kalamazoo, Michigan49008, United States; § Department of Nanoscience, Joint School of Nanoscience and Nanoengineering, University of North Carolina at Greensboro, 2907 East Gate City Boulevard, Greensboro, North Carolina27401, United States; ∥ Department of Nanoengineering, Joint School of Nanoscience and Nanoengineering, North Carolina A&T State University, 2907 East Gate City Boulevard, Greensboro, North Carolina27401, United States

## Abstract

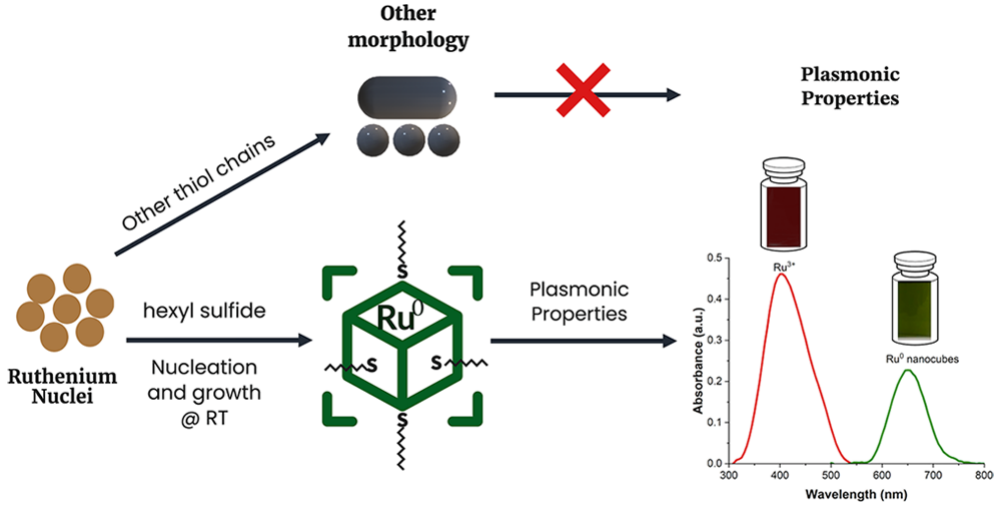

Controlling the nucleation and growth processes for nanoparticle synthesis allows the development of well-defined structures that offer unique chemical and physical properties. Here, we report a wet chemical reduction method for synthesizing ruthenium nanocubes (Ru NCs) that display plasmonic properties at room temperature (RT). The growth of the particles to form nanostructured cubes was established by varying the carbon chain length of the thioether stabilizing ligands and the reaction time to produce stable and controlled growth. In this study, we found that the longer the thioether chain length, the less isotropic the shape of the particles. Short chain lengths of thioethers (ethyl sulfide and butyl sulfide) produced spherical nanoparticles, whereas longer chain lengths (hexyl sulfide and octyl sulfide) produced cubic nanoparticles. In addition, parameters such as the ligand to precursor ratio also played an important role in the homogeneity of the nanocubes. The Ru NCs were characterized by UV–visible absorbance spectroscopy, transmission electron microscopy (TEM), X-ray diffraction (XRD), and X-ray photoelectron spectroscopy (XPS), which supported a face-centered cubic (fcc) structure. Moreover, to demonstrate catalytic efficiency, we studied their ability to reduce benzaldehyde to benzyl alcohol, and the Ru NCs demonstrated an overall 78% efficiency at room temperature.

## Introduction

In recent years, research has been devoted to the development of new materials with properties that can be controlled as a result of unique size, shape, configurations, and morphology.^1−4^ In the case of nanoscale particles, attaining structures with well-defined size or shape leads to properties that are often superior to those of bulk materials. Consequently, methods and procedures that result in anisotropic nanostructures are desirable, especially if the process to design and produce them is followed in a way that minimizes energy utilization, for example, room-temperature-based procedures. Metal nanoparticles can be controlled by varying parameters involved in the reaction, including metal salt precursors, the ligand/stabilizer, the reductant, the reaction duration, and the reaction temperature. Attractive chemical and physical properties are exhibited when the shape and size of metallic nanostructures are manipulated. For example, optical, catalytic, electrochemical, magnetic, and even surface energy properties may be altered when a metal nanoparticle’s size or shape is modified. While controlling the shape of metallic nanomaterials has presented challenges in the past, substantial progress has been made in establishing synthetic methods for well-defined anisotropic nanoparticles consisting of metal (Au, Ag, Pd, and Pt) nanostructures.^5−8^

Ruthenium nanoparticles are of great interest due to their catalytic properties and cost relative to those of the other metals. Although Ru is a promising d-group metal, due to its broad oxidation states, cost, and catalytic behavior, stable nanostructures with controlled morphology are limited.^9−12^ Recent studies have discovered that Ru on the nanoscale can grow in hexagonal cubic packing (hcp) and face-centered cubic packing (fcc) crystal structures, providing access to controlling the size and morphology that are stable with enhanced performance.^13^ The fcc Ru NPs, which are available only on the nanoscale, have displayed a higher catalytic response than hcp due to the presence of the (111) plane at lower energy.^14,15^ Nevertheless, limited studies have been able to explore these properties successfully. Seed-mediated synthesis of ruthenium nanoparticles has been explored over the years, allowing the diverse growth pattern and development of nanostructures. Ye et al. used a Pd seed to produce fcc Ru nanoframes through seeded growth and chemical etching.^16^ Zhao et al. used Rh NCs or Pd NCs as a seed for the growth of Ru octahedral nanocrystals that led to the formation of stable nanostructures at elevated reaction temperatures.^15,17^ Polyol synthesis is one of the popular methods that involves glycol that serves as a solvent and reducing agent in a reaction that controls the uniformity of sizes.^18^ These synthetic routes have been promising and display higher catalytic activity. However, there is often a requirement for harsh reaction conditions that lead to oxidative etching of the seed, lack of nanostructure, or the formation of an alloy complex.

Herein, we have developed a facile reduction method to produce plasmonic ruthenium nanocubes (Ru NCs). We have aimed to understand the growth pattern of ruthenium active sites toward the production of stable nanocube structures. Controlling the growth of nanostructures often relies on the ligand species and reducing agents that influence shape control and the stability of nanostructures. The thiol ligand’s high binding affinity for Pt-group metals such as Ru can be promising for controlling directional growth.^3,18,19^ The presence of these materials may also influence contamination and growth at the surface level.

We have investigated the influence of the ligand carbon chain length and reaction time on the growth of Ru NCs. To our knowledge, this is the first article to produce plasmonic Ru NCs following a facile procedure at room temperature. Exploring the growing influence of thiol ligands and reducing agent NaBH_4_ at room temperature, we demonstrated that fcc Ru NCs could be produced with a uniform shape and size distribution. A number of characterization techniques including transmission electron microscopy (TEM), X-ray diffraction (XRD), selected area electron diffraction (SAED), and UV–visible absorbance spectroscopy were used to study the characteristics of the Ru nanostructures. In addition, we also investigated the potential catalytic behavior of thioether-stabilized Ru nanostructures in the reduction of benzaldehyde to benzyl alcohol.

## Experimental Section

### Materials

Ruthenium(III) chloride hydrate [RuCl_3_·H_2_O], ruthenium(III) bromide hydrate [RuBr_3_·H_2_O], and ruthenium(III) acetate were purchased from Strem Chemicals. Hexyl sulfide, ethyl sulfide, butyl sulfide, octyl sulfide, ethanol, hydrazine hydrate, and sodium borohydride were purchased from Sigma-Aldrich with no further purification. All solvents (Sigma-Aldrich) were of HPLC grade or better and were distilled prior to use.

### Synthesis of Ru NCs

RuCl_3_·H_2_O (0.1 g, 0.49 mmol) was dissolved in 20 mL of ethanol. Hexyl sulfide (0.996 g, 4.9 mmol) and sodium borohydride [NaBH_4_] (0.0186 g, 0.49 mmol) were added to the solution. The preferred ratio of the metal precursor to the stabilizer was 1:10. The resulting solution was allowed to sit undisturbed under vacuum at room temperature for 5 h. The color of the solution changed from dark orange to dark green.

### Synthesis of Ru NCs with Varying Ratios of Hexyl Sulfide

The reaction synthesis was also carried out for metal precursor to stabilizer ratios of 1:5, 1:15, and 1:20. RuCl_3_·H_2_O (0.1 g, 0.49 mmol; 0.1 g, 0.49 mmol; and 0.1 g, 0.49 mmol) in the case of varied ratios 1:5, 1:15, and 1:20, respectively, was dissolved in 20 mL of absolute ethanol. The stabilizing ligand (hexyl sulfide: 0.5 g, 2.5 mmol; 1.5 g, 7.3 mmol; and 2.0 g, 9.7 mmol) and reducing agent (NaBH_4_: 0.0186 g, 0.49 mmol; 0.0186 g, 0.49 mmol; and 0.0182 g, 0.49 mmol) were added to the solutions. Again, these reaction mixtures were allowed to sit undisturbed under vacuum. After 5 h, a 1 μL aliquot of each solution was removed to prepare grids for TEM imaging.

### Synthesis of Ru NCs with Varying

Thioether-stabilized Ru nanoparticles were generated using ethyl sulfide, butyl sulfide, and octyl sulfide. RuCl_3_·H_2_O (0.1 g, 0.49 mmol; 0.49 mmol; and 0.49 mmol) was dissolved in 20 mL of ethanol in three Erlenmeyer flasks. The stabilizing ligands ethyl sulfide, butyl sulfide, and octyl sulfide (0.427 g, 4.7 mmol; 0.713g, 4.8 mmol; and 1.255 g, 4.8 mmol) were added to the solutions, respectively. All solution colors were dark orange at the beginning of the reaction. The flasks were capped with a rubber septum and stored in a vacuum desiccator. After 5 h, a 1 μL aliquot of each solution was removed to make a grid for TEM imaging.

### Characterization

Transmission electron microscope (TEM) images were obtained using a JEOL electron microscope, model JEM-1230. High-resolution transmission electron micrographs and SAED images were obtained using a JEOL 2100PLUS high-resolution transmission electron microscope (HRTEM). Powder X-ray diffraction (XRD) data was collected on a Scintag XDS model 2000 diffractometer. For XRD measurements, the Ru nanoparticle sample was dried and mixed with 325 mesh Si powder and was placed on a Si wafer sample holder. To begin the drying process for XRD analysis, the sample is transferred to a vacuum oven and allowed to sit overnight. To complete the drying process, the flask was placed in a silicon oil bath at a temperature of 230 °C for 20 min under a flow of nitrogen to ensure that the reaction mixture was free from all solvents. The resulting solid residue was scraped from the flask using a scoopula and packed into a 0.3 mm boron-rich glass X-ray capillary tube and then used for XRD analysis. The X-ray photoelectron spectroscopy (XPS) was conducted for a drop-casted sample on a Si wafer, and the surface species analysis was performed using the XPS/UVS – SPECS system with a PHOIBOS 150 analyzer.

### Catalytic Measurements

The reaction mixture contained 0.5 mL of benzaldehyde, 0.2 g of Ru/SiO_2_ nanomaterials, and 10 mL of ethanol in a 50 mL round-bottomed flask capped with a rubber septum. Hydrogen gas (H_2_(g)) was bubbled into the reaction solution for 8 h while stirring at room temperature. Afterward, the solution was refluxed in a silicon oil bath at 85 °C under an H_2_(g) atmosphere.

## Results and Discussion

**Scheme 1:**
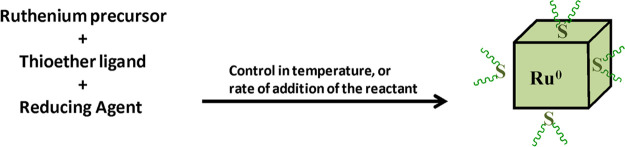
Schematic Illustrating the Synthesis of Ruthenium Nanocubes at Room Temperature

To provide an inert atmosphere, cubic Ru nanoparticles were synthesized in an Erlenmeyer flask capped with a rubber septum. RuCl_3_ was dissolved in 20 mL of absolute ethanol. Hexyl sulfide was added to this solution as a stabilizing ligand, and the reaction mixture was allowed to stir for approximately 5 min. Scheme 1 provides a visual description of the formation of Ru NPs. The solution was a dark orange-brown color before the reducing agent was added. Sodium borohydride was used as a reductant and was added dropwise to the reaction mixture. The solution had a slight bubbling effect upon adding the sodium borohydride, indicating reduced Ru^3+^ to Ru^0^.

The resulting solution was allowed to sit undisturbed at room temperature in a vacuum desiccator for the first 5 h, saving the nanoparticle surface from being oxidized and possibly changing the structure.^20^ This is done to prevent oxidative etching, a process whereby zero-valent metal atoms are oxidized back to ions (i.e., Ru^0^ → Ru^3+^).^21^ A 1 μL aliquot of this solution was removed to prepare a 400 mesh Formvar-coated (ultrathin carbon type) copper grid for TEM, HRTEM, and SAED analysis.

Previous studies using solution-based synthesis with ruthenium precursors have found it challenging to prepare nanoparticles with identifiable shapes and sizes.^21^ Zhao et al. developed different Ru nanostructures using the seeded growth method followed by a chemical etching process. They have produced Ru cubic nanocages using seed-mediated synthesis by controlling the reaction temperature and the deposition rate of metal precursors.^22^ The template effect of Pd nanocubes acts as seeds to deposit Ru atoms layer-by-layer on the Pd cubes and finally yield the fcc structure of Ru cubic nanocages. Ye et al. fabricated Ru nanoframes with fcc structure obtained by the epitaxial crystal growth of Pd seeds to the Ru surfaces due to the driving force of reducing the total surface energy of nanostructures.^16^ Another study reported the synthesis of Ru octahedral nanocrystals using the Rh nanocube seeds at high temperatures.^15^ These studies rely on the template seeds of Pd or Rh that have an fcc structure to facilitate epitaxial crystal growth on the surface in order to deposit Ru atoms and yield an fcc structure with different morphologies. In contrast, we were able to define the morphology of our ruthenium nanoparticles distinctly at room temperature, without the use of a metal template, with fcc structure. To generate zero-valent atoms when synthesizing metal nanomaterials, the precursor compound may follow one of two pathways: (1) decomposition or (2) reduction.^23^ Normally, decomposition of the precursor occurs during the synthesis when the solution is exposed to heat or sonication in which aggregation of the atoms forms nuclei (self-nucleation).^23,24^ The reduction of a metal precursor compound can occur via two pathways: (1) by direct conversion to nuclei and then addition to the existing precursor-based nuclei or (2) by growing nanoparticles without reaching a zero-valent oxidation state.^23^

**Figure 1:**
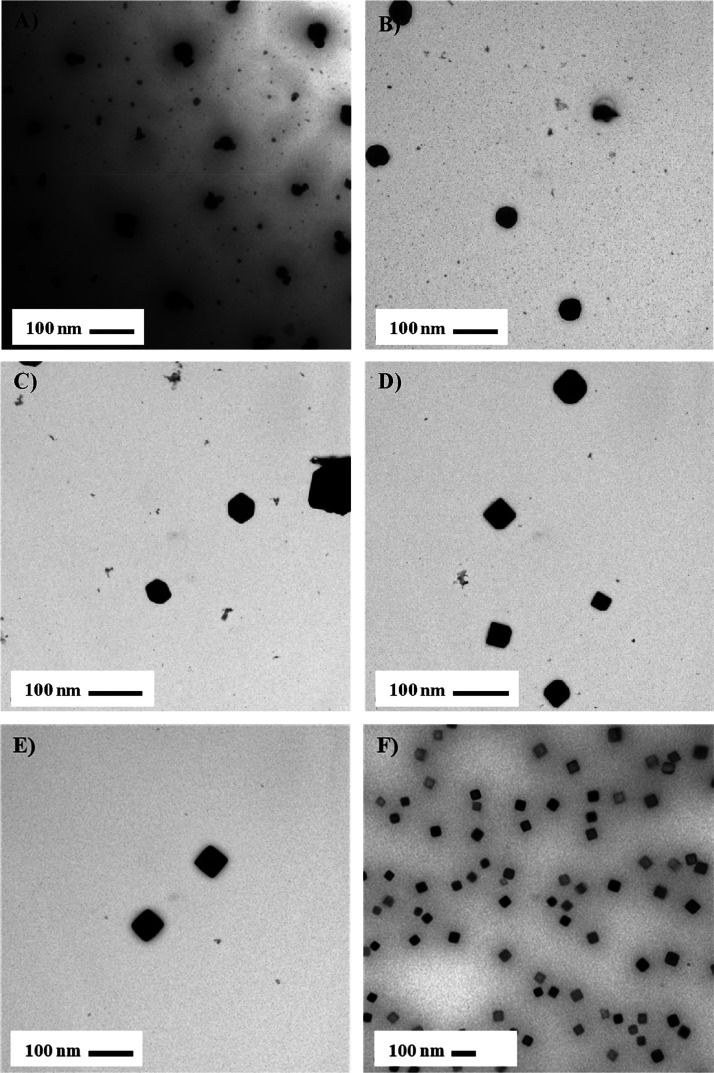
TEM images of the metal precursor to stabilizing ligand (1:10) synthesized Ru nanoparticles in hexyl sulfide during time progression analysis. (A) 0, (B) 1, (C) 2, (D) 3, (E) 4, and (F) 5 h.

A time progression analysis was completed for the 1:10 (metal precursor to stabilizing ligand) ratio of the Ru nanoparticles to better understand the growth mechanism. Figure 1A–F depicts the growth of the Ru nanoparticles over a 5 h time period and captures the structural changes. At the beginning of the reaction (<1 min after the addition of the reducing agent), the particles maintain a spherical shape (Figure 1A,B). As the reaction proceeds through the second, third, and fourth hours (Figure 1C–E), we begin to see changes in the morphology (cubic structure).

**Figure 2:**
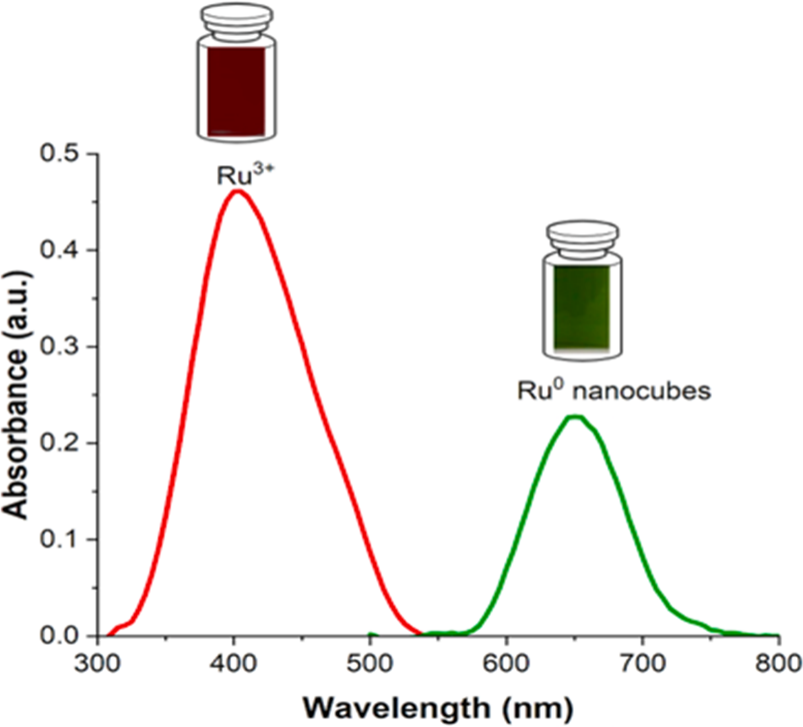
UV–visible absorbance spectra of RuCl_3_ in ethanol (red line) and of RuCl_3_ with hexyl sulfide (1:10) in ethanol after 5 h (green line) indicating the formation of cubic Ru nanoparticles with an absorbance intensity peak at 395 and 655 nm, respectively.

The Ru nanoparticles appear to have a hexagonal shape after 2 h of reacting (Figure 1C). Figure 1D exhibits cubic nanoparticles that are truncated. The first evidence of cubic Ru nanoparticles with sharp edges and corners is illustrated in Figure 1E. Figure 1F shows well-defined Ru nanocubes stabilized with hexyl sulfide (1:10) depicted with an edge length of 50–80 nm. It can be seen in the UV–visible absorbance spectrum that RuCl_3_ in ethanol alone has an intensity peak at 395 nm (Figure 2). It indicates the Ru^3+^ form in the solution. After the stabilizing ligand (hexyl sulfide) is added and the reaction solution is allowed to sit for 5 h, the absorbance peak shifts to a longer wavelength (655 nm). Although cubic Ru nanomaterials have not shown a plasmonic peak,^25^ the synthesis explained here shows the plasmonic properties that suggest Ru^3+^ has been completely reduced and Ru nanocubes stabilized with thioethers were formed.

This longer-wavelength shift is associated with surface plasmon resonances (SPR) of produced Ru nanostructures. Noble metals such as Ru have plasmonic properties, and longer wavelengths have resulted from increasing the size of produced self-assembled 3D nanocubes from the initial metal atoms. Therefore, it further confirms that there is a change in the nanomaterials’ size, shape, and/or morphology from the initial metal precursor. The inset in Figure 2 depicts the Ru nanocubes’ final solution color (green).

According to Viau et al., ruthenium nanoparticles have been synthesized using RuCl_3_ as a precursor and 1,2-propanediol as a stabilizer.^3,26,27^ As the reaction progressed, the color of the solution changed from red to green and finally to dark brown. This change in color indicates the transformation of the size and/or shape of the particle.

**Figure 3:**
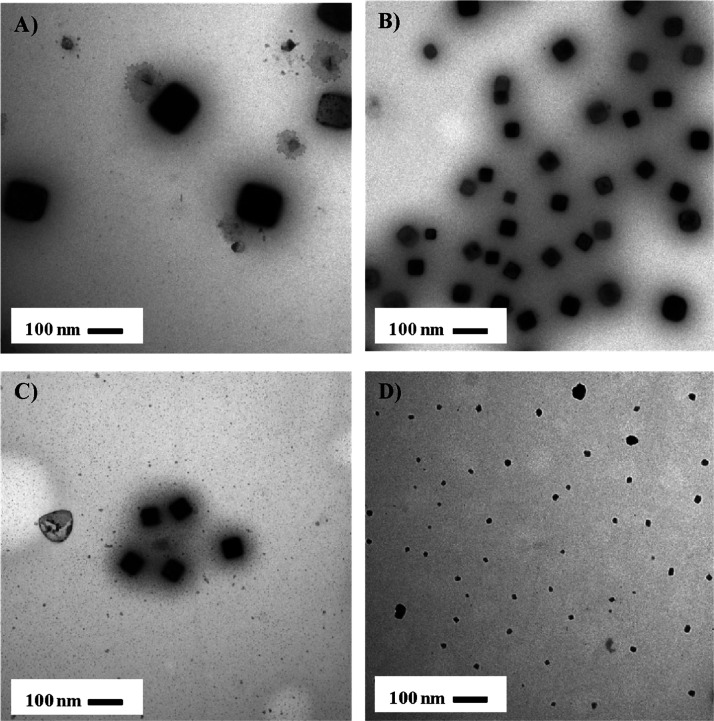
TEM image of the metal precursor to ligand (A) 1:5, (B) 1:10, (C) 1:15, and (D) 1:20 synthesized Ru nanocubes in hexyl sulfide.

Thioethers have yet to be fully exploited as stabilizers or size adjusters for the fabrication of metallic nanomaterials except for several research groups.^28−32^ Thioethers behave as soft donors, as do phosphine ligands. Nonetheless, thioethers are less toxic and fairly easy to handle in oxygenated environments.^28^ In this study, we investigated several thioether ligands (ethyl sulfide, butyl sulfide, hexyl sulfide, and octyl sulfide) as stabilizers while observing the shape and size variations with respect to the thioether carbon chain length. TEM images of the Ru nanoparticles stabilized in ethyl sulfide, butyl sulfide, and octyl sulfide can be found in Supporting Information (Figure S1). We found that there was significant control over the shape of the Ru nanoparticles as the thioether carbon chain length increased. In the case of ethyl sulfide, cubic morphology is not distinguishable. Butyl sulfide provides an illustration of Ru nanoparticles that have not been fully formed into the cubic structure, whereas octyl sulfide shows a cubic formation with truncated corners.^3,18,19^ Corresponding ratios of the nanocubes using hexyl sulfide as the stabilizing ligand are illustrated in Figure 3A–D.

As the varying amount of thioether ligand changes, so does the size of the nanoparticle. When the molar ratio of hexyl sulfide is 5 times that of the ruthenium precursor, the size of the nanoparticles is 100–150 nm (Figure 3A). It can be shown in Figure 3B,C that as the ratio of the ligand increases, the size of the nanoparticle decreases. These two ratios have similarly sized nanocubes. Synthesizing the Ru nanoparticles with a molar ratio of the ligand 20 times that of the precursor did not result in nanoparticles with a cubic structure, and the size has decreased up to 10–30 nm.

**Figure 4:**
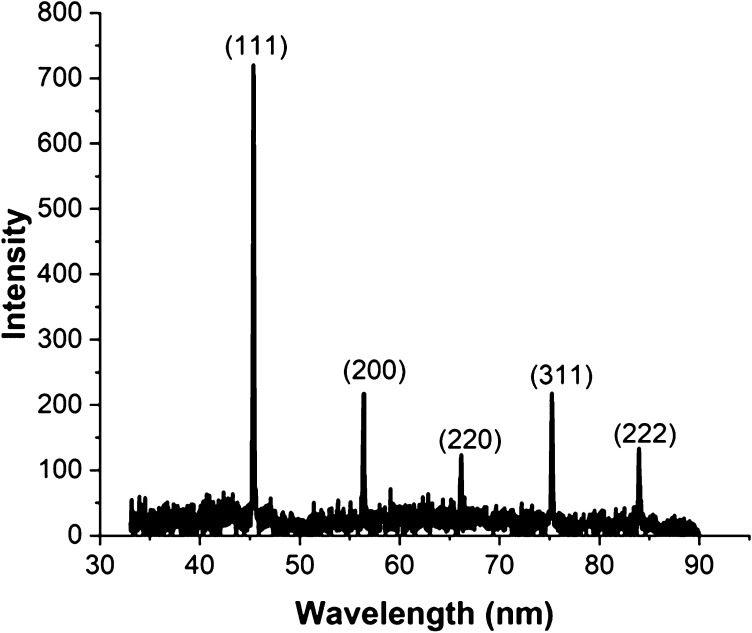
Powder X-ray diffraction patterns of Ru nanocubes.

Many of the metals tend to adopt one of the three basic crystalline structures: body-centered cubic (bcc), face-centered cubic (fcc), or hexagonal close-packed (hcp). The structural stability of bulk metal materials has been extensively investigated.^13,33−35^ Bulk ruthenium is known to take an hcp crystal structure; however, on the nanoscale, ruthenium can form an hcp or fcc structure. X-ray diffraction measurements are ideal for determining the structure of metal nanoparticles such as Pt,^36^ Au,^36^ Ag,^37^ Cu,^38^ Pd,^39^ and Zn.^40^ The XRD patterns of the Ru nanocubes are shown in Figure 4.

**Figure 5:**
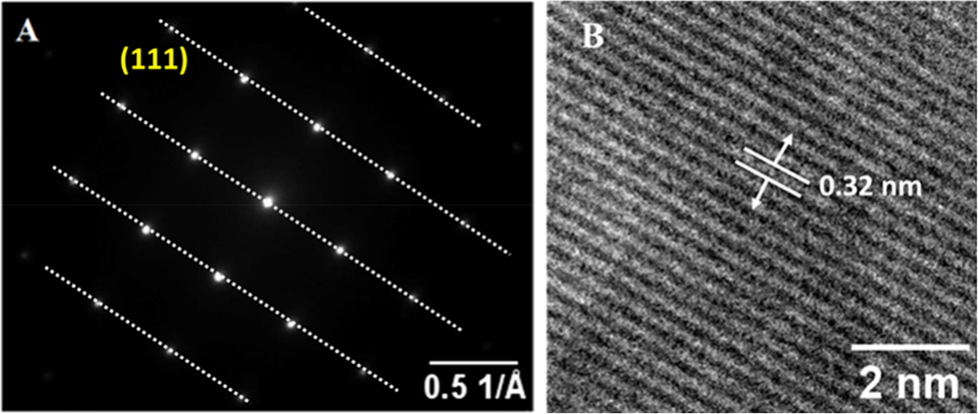
(A) SAED and (B) HRTEM images of ruthenium nanocubes having a crystal spacing of 0.32 nm.

The Bragg angle for the cubic Ru nanomaterials is 2θ = 45.567, 56.484, 66.241, 75.308, and 84.011°, corresponding to the Ru(111), (200), (220), (311), and (222) diffraction patterns, respectively. The peak intensity heights for diffraction planes are supported to reflect the (111) crystal facet and reveal the favorable direction for Ru nanocube growth and provide evidence for the formation of fcc crystal structure. Similar evidence of fcc Ru nanoparticles was reported by Kitagawa et al.^13^ Selected-area electron diffraction (SAED) also confirms that the structure of Ru nanocubes is single-crystalline (Figure 5A). High-resolution TEM studies also support that the lattice fringe spacing of the Ru NCs is 0.32 nm, as shown in Figure 5B, and can be assigned to the {111} planes of fcc.

The shape of a metallic nanoparticle can vary based on the solvent utilized.^41−44^ In 2007, Chu et al. were able to tune the shape of indium oxide (In_2_O_3_) nanomaterials by varying the solvent from methanol to ethanol.^41^ In their study, methanol as a solvent produced cubic In_2_O_3_ particles while ethanol produced rods.^41^ Yuan et al. developed rhodium nanocrystals with varying morphologies such as cubes, dendrites, and horned particles through a synergetic effect of sodium lauryl sulfate and halogen anions using water as the solvent.^43^

**Figure 6:**
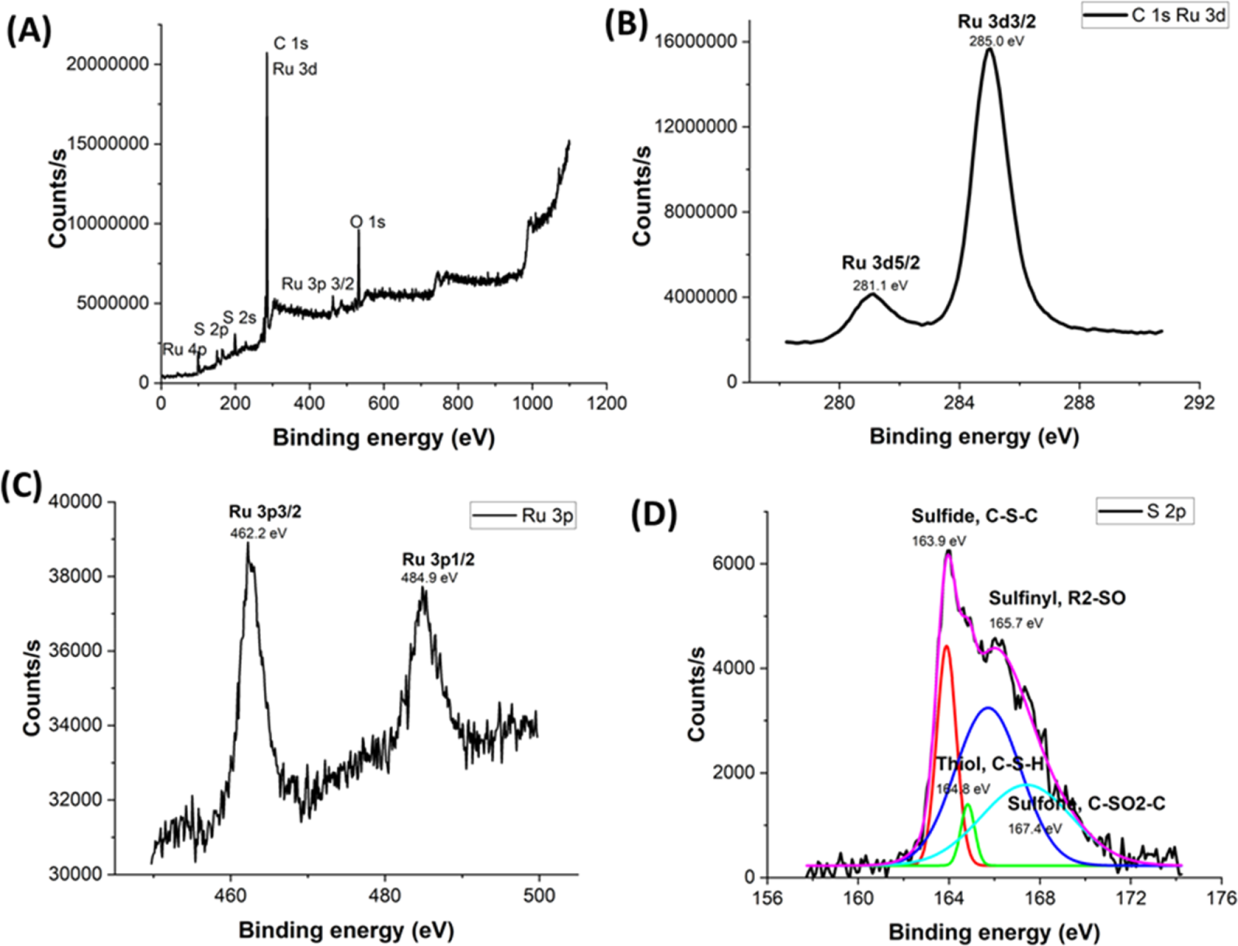
XPS spectra of ruthenium nanocubes: (A) the survey XPS spectrum and the binding energy spectra of (B) C 1s, Ru 3d, (C) Ru 3p, and (D) S 2p.

The surface species and oxidation states for the synthesized Ru NCs were elucidated by X-ray photoelectron spectroscopy (XPS). The XPS survey spectrum depicted in Figure 6A confirms the presence of Ru, S, and C in these nanocubic structures. The peaks in the high-resolution spectrum within Figure 6B demonstrated that there is a strong overlap between the Ru 3d and C 1s regions, and their binding energy spectrum exhibits two peaks at 281.1 and 285 eV, which are characteristic of the binding energy states of Ru 3d_5/2_ and Ru 3d_3/2_, respectively. Therefore, the Ru 3p spectrum (Figure 6C) was investigated further instead of Ru 3d, and it has shown that there are two peaks for the binding energy spectra of Ru 3p at 462.2 and 484.9 eV which correspond to Ru 3p_3/2_ and Ru 3p_1/2_, respectively. In past studies, it has been reported that binding energies of Ru 3p exhibit two peaks when there is a presence of metallic ruthenium.^45−47^ Furthermore, this observation can confirm the formation of the Ru^0^ metallic form (Table S1). The sulfur binding energy spectrum shows four peaks of S 2p_3/2_ at 163.9, 164.8, 165.7, and 167.4 eV, which can be assigned to the unreacted sulfide, thiol group, sulfinyl, and sulfone groups, respectively.^48−50^ Additionally, the O 1s spectrum (Figure S3) shows a peak centered at 532.5 eV which depicts the absorbed oxygen.^51^ Moreover, this confirms that there are no metal oxides or hydroxides being formed.

To examine the effects of different solvents and/or solvent ratios on controlling the shape of the Ru nanoparticles, Milli-Q (18.2 MΩ at room temperature) water was utilized in conjunction with ethanol. Figure S2 depicts the TEM images for the reaction solutions for Ru nanoparticles that contain various ratios of MQ H_2_O and ethanol. When 20 mL of MQ H_2_O was used as the solvent in the reaction mixture, the TEM image (S2 (A)) revealed Ru particles that had a spherical shape. Figure S2(B) (80:20 MQ H_2_O/ethanol) revealed Ru particles that had a mixture of spherical and cubic morphology. The Ru nanoparticles illustrated in Figure S2(C), which contains 10 mL of MQ H_2_O and 10 mL of ethanol (50:50), disclosed particles with a somewhat irregular cubic shape. Ru nanoparticles with a rod-like shape can be seen in Figure S2(D). Here, only 4 mL of MQ H_2_O is used with 16 mL of ethanol (20:80).

Ruthenium is very important in metallic materials science because of its attractive catalytic activity. Ru NPs are effective materials in catalysis for various chemical reactions that include deoxygenation, hydrogenolysis, and hydrogenation.^52,53^ In 2010, Julis et al. conducted a hydrogenation reaction of biomass-derived substrates in ionic liquids (IL) using Ru nanoparticles.^52^ They found that the Ru NPs were effective catalysts for the hydrogenation reaction. Controlling the variation of the IL will affect the activity, selectivity, and stability of the nanoparticles.

**Figure 7:**
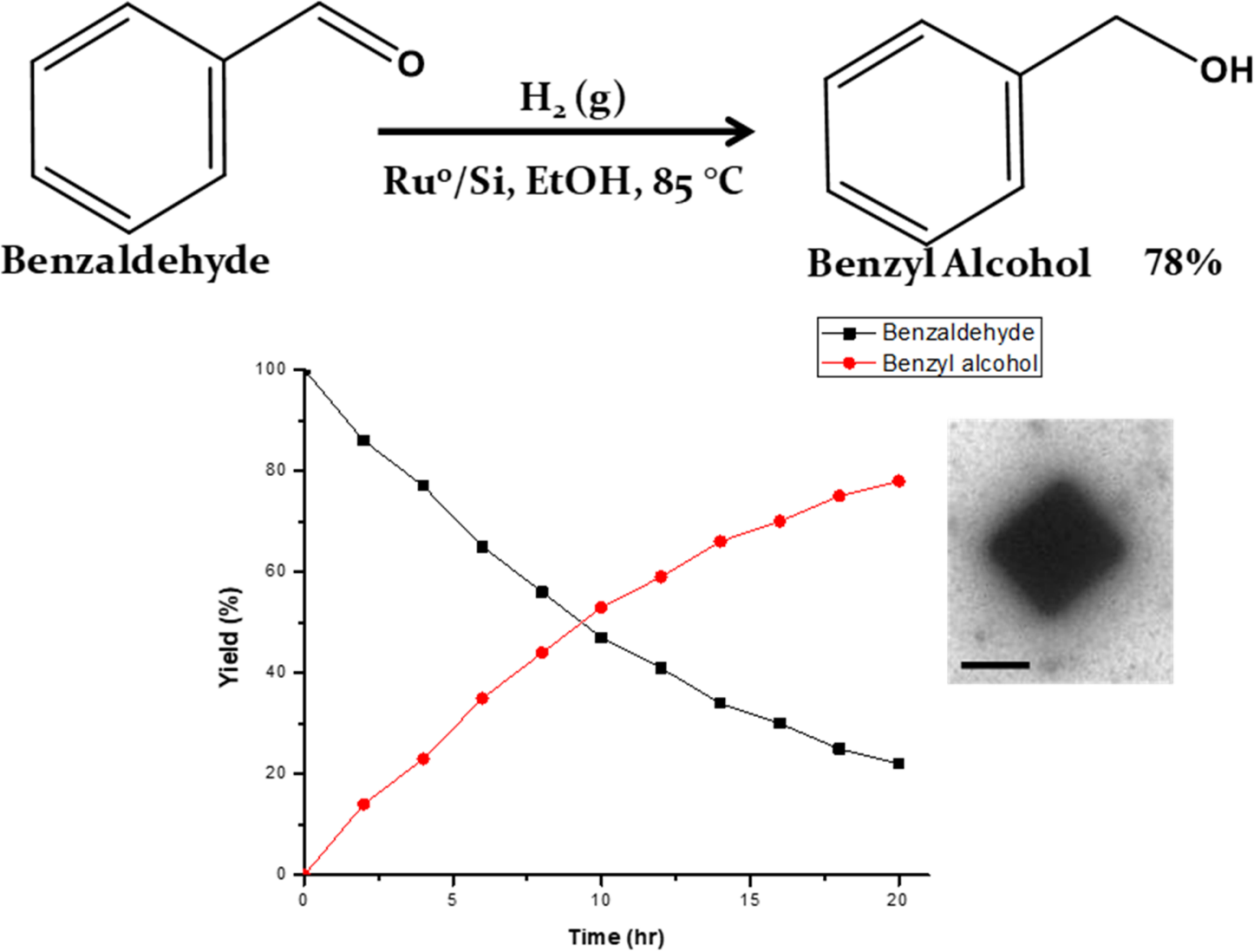
Reaction scheme for the conversion of benzaldehyde to benzyl alcohol along with a graph that represents the percent yield versus time. The TEM image is of a Ru nanoparticle supported on silica. The scale bar is 100 nm.

For the catalytic hydrogenation of benzaldehyde to benzyl alcohol systems, metals (Ru, Pd, Pt, Cu, Co, and Ni) may be supported on various materials such as SiO_2_, Al_2_O_3_, ZrO_2_, CeO_2_, TiO_2_, mesopolymers, and carbon nanomaterials.^54−61^ The choice of the support is of importance because the support affects the metal, the adsorption of the reagents, and the mass transfer processes.^62^ Metal supports also play a fundamental role in determining the catalyst activity and selectivity. In this project, silica gel (SiO_2_) was used as the support to disperse and stabilize the Ru nanocubes (Figure 7). Ru-based catalysis has lower reactivity than other noble metals due to the higher reduction of the carbonyl group of ketones and aldehydes. The work here illustrates the effects of the silica gel and ruthenium metal for the hydrogenation of benzaldehyde to benzyl alcohol in ethanol. As a commercially important chemical, benzyl alcohol has been used in several technological areas such as pharmaceuticals, industry, and nanotechnology.^63,64^ Much attention has been paid to the catalytic hydrogenation of benzaldehyde for the production of benzyl alcohol, and this investigation has demonstrated the potential applications of the synthesized Ru NCs with thioether groups.

## Conclusions

We have shown that thioethers play a significant role in the synthesis and stabilization of ruthenium nanoparticles and how chain length influences the particle shape. Previous synthetic strategies used to produce cubic-shaped Ru nanoparticles required the use of templates and high temperature. Here we demonstrate a procedure that is easily applicable to obtaining well-defined nanocubes of ruthenium under room-temperature conditions. The established facile synthetic method for producing ruthenium nanocubes leads to nanocubes with an edge length of 50–80 nm. More importantly, by varying the ratios of the reactants one can control the morphology of the nanomaterials. The ability to produce nanoparticles using parameters that minimize energy utilization are especially attractive for future industrial applications and for the scale-up of reactions. Furthermore, controlling the shape of metal nanomaterials is important because it allows one to tune the properties, such as optical, magnetic, electronic, and catalytic, for various applications. The notable feature is the observed optical properties of the nanocubes, and the investigation of this feature will be the subject of future studies in our laboratory.

## Supporting Information Available

Additional TEM images of the synthesis using different thiol ether stabilizing ligands and varying ratios of MQ H_2_O and ethanol that produce different morphologies of Ru nanostructures; additional information on XPS analysis (PDF)
