# Supplementary material for: Room-Temperature Synthesis of Thioether-Stabilized Ruthenium Nanocubes and Their Optical Properties
Source: Langmuir. 2023 Feb 1;39(7):2500–8. doi: 10.1021/acs.langmuir.2c02645 (PMC9948292; doi:10.1021/acs.langmuir.2c02645)
Supplement: Supplementary file 1 — la2c02645_si_001.pdf [file la2c02645_si_001.pdf]

Supporting Information

Room-temperature Synthesis of Thioether-stabilized Ruthenium Nanocubes and their Optical Properties

*Clara P. Adams[1,4]; Chartanay D. J. Bonner[2,4]; Gayani Pathiraja[2]; Sherine O. Obare[2,3,4]**

[1]Central Piedmont Community College, 1201 Elizabeth Avenue, Charlotte, NC 28204, USA

[2]Department of Nanoscience, Joint School of Nanoscience and Nanoengineering, University of North Carolina at Greensboro, Greensboro, NC 27401, USA

[3]Department of Nanoengineering, Joint School of Nanoscience and Nanoengineering, North Carolina A&T State University, Greensboro, NC 27401, USA

[4]Department of Chemistry, Western Michigan University, 1903 W. Michigan Ave. Kalamazoo, MI 49008

Corresponding Author:

*Email: soobare@ncat.uncg.edu Tel : +1-336-285-2805

TEM images of ruthenium nanoparticles synthesis using different thiol ether stabilizing ligands, varying ratios of MQ H_2O and ethanol that produce different morphologies …………………..………………………………………………………………………2

XPS analysis of ruthenium nanocubes ….………………………………………………………4

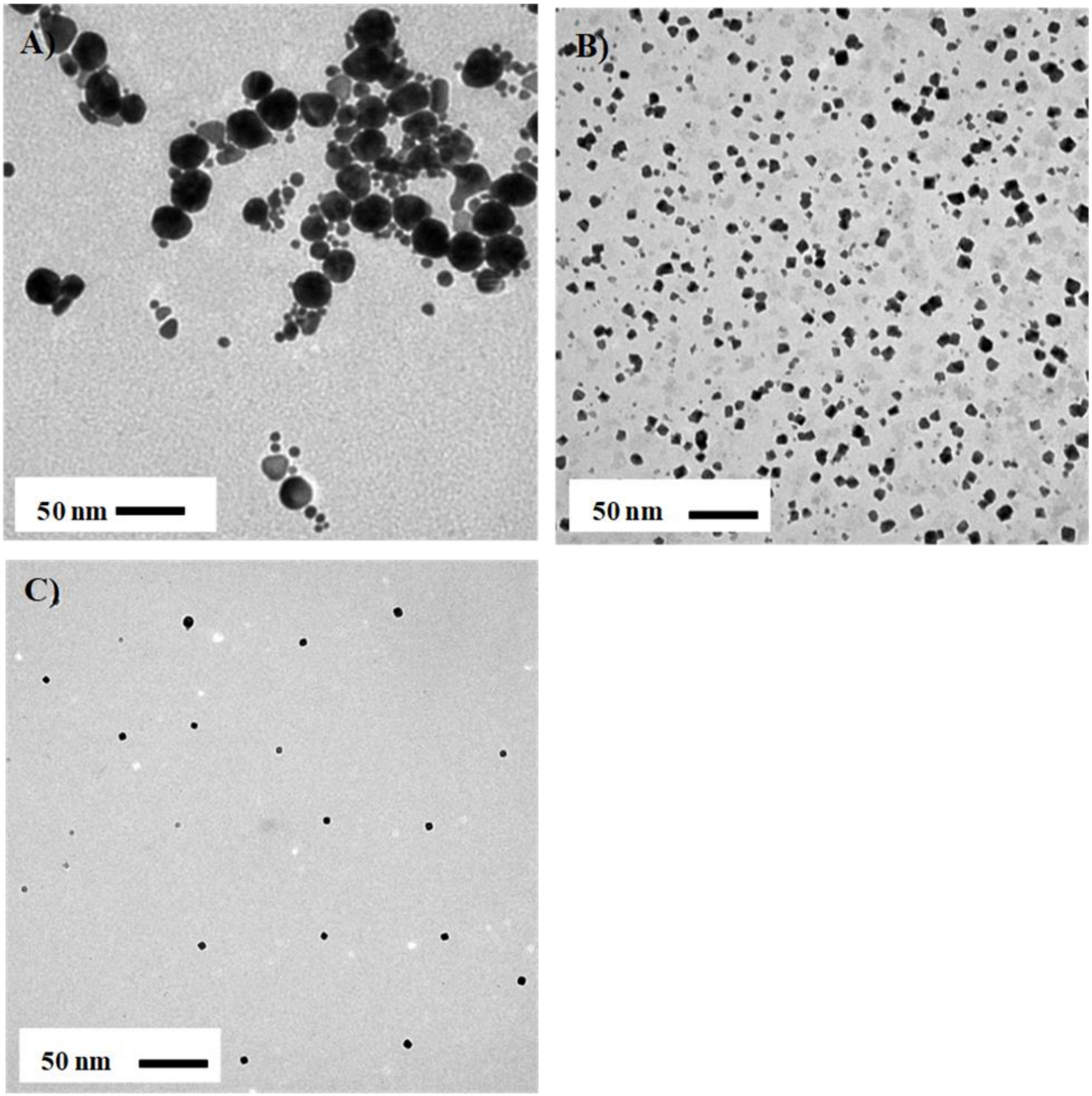

Figure S1 : TEM images of Ru nanoparticles stabilized in (A) ethyl sulfide, (B) butyl sulfide and (C) octyl sulfide.

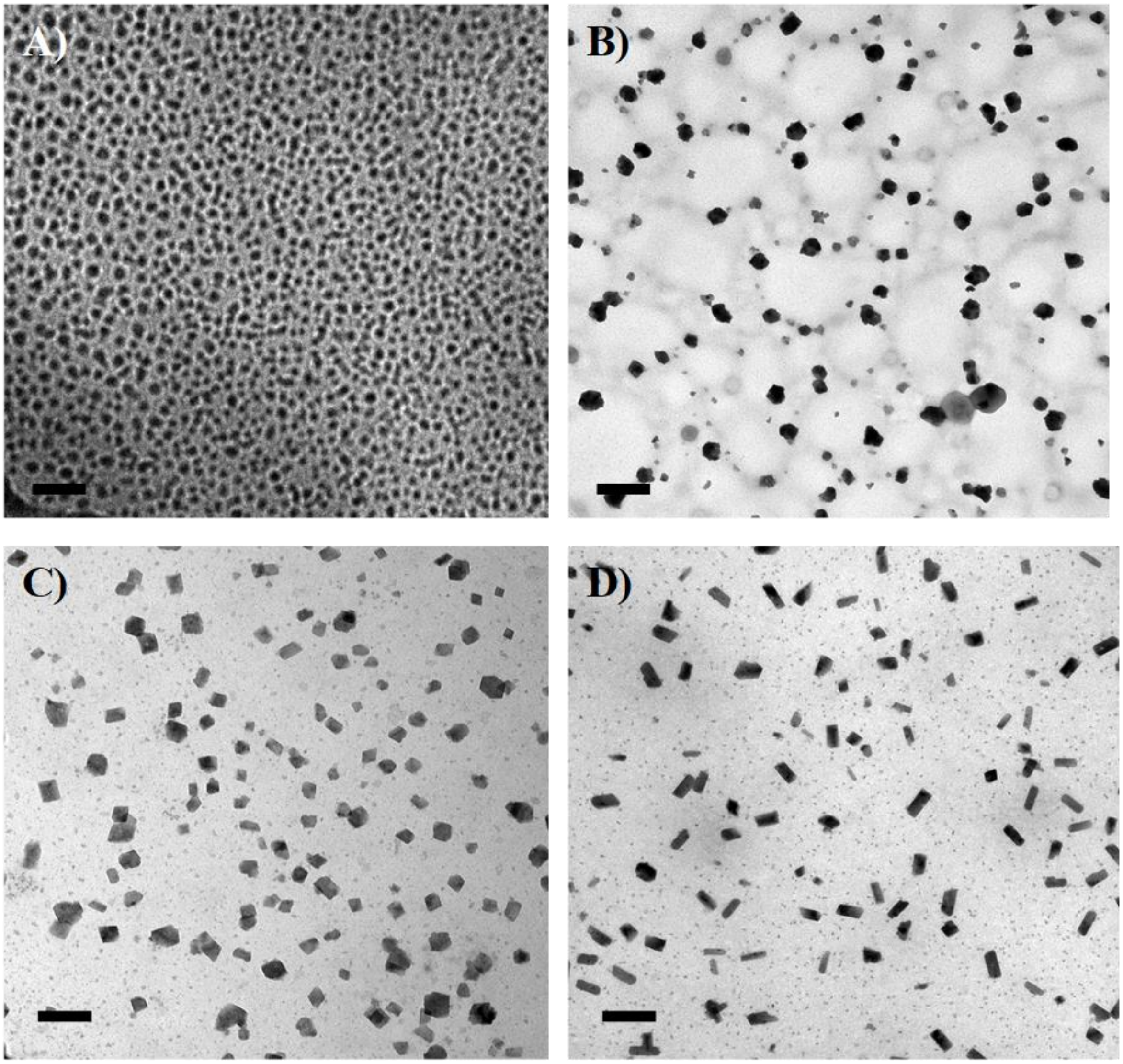

Figure S2: TEM images of Ru nanoparticles with varying ratios of MQ H_2O and ethanol to produce different morphologies. (A) 100% of MQ H_2O, (B) 80% of MQ H_2O and 20% of ethanol, (C) 50% of MQ H_2O and 50% of ethanol, and (D) 20% of MQ H_2O and 80% of ethanol. The scale bar represents 100 nm.

Table S1: XPS analysis of synthesized Ru NCs using the hexyl sulfide as stabilizing ligand with the molar ratio of 1:10 after 5 hours.

Element	Peak	Position BE (eV)±0.1 eV	FWHM (eV) ±0.20 eV	Atomic Con. (%)
Ru	Ru $3P_{3/2}$; Ru $3P_{1/2}$	462.2, 484.9	2.90	28.9
S	S $2P_{3/2}$	163.9, 164.8, 165.7, 167.4	3.49	71.1

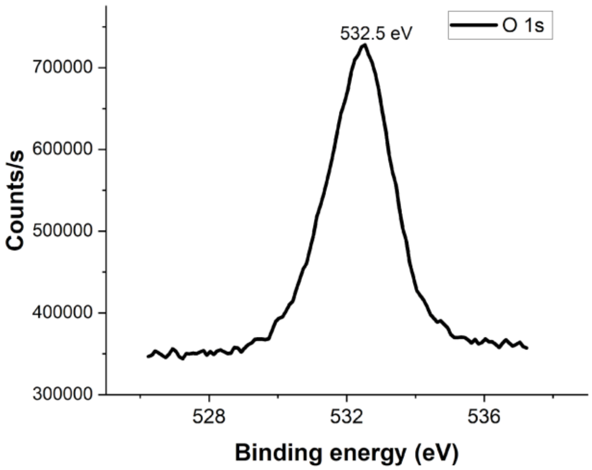

Figure S3: XPS binding energy spectra of O 1s for Ru NCs.
